# Duck gut metagenome reveals the microbiome signatures linked to intestinal regional, temporal development, and rearing condition

**DOI:** 10.1002/imt2.198

**Published:** 2024-05-14

**Authors:** Lingyan Ma, Wentao Lyu, Tao Zeng, Wen Wang, Qu Chen, Jiangchao Zhao, Guolong Zhang, Lizhi Lu, Hua Yang, Yingping Xiao

**Affiliations:** ^1^ State Key Laboratory for Managing Biotic and Chemical Threats to the Quality and Safety of Agro‐products, Institute of Agro‐product Safety and Nutrition Zhejiang Academy of Agricultural Sciences Hangzhou China; ^2^ Institute of Animal Husbandry and Veterinary Medicine Zhejiang Academy of Agricultural Sciences Hangzhou China; ^3^ Department of Animal Science, Division of Agriculture University of Arkansas Fayetteville Arkansas USA; ^4^ Department of Animal and Food Sciences Oklahoma State University Stillwater Oklahoma USA

**Keywords:** duck, gastrointestinal microbiome, intestinal regional, metagenome‐assembled genomes, rearing condition, temporal development

## Abstract

The duck gastrointestinal tract (GIT) harbors an abundance of microorganisms that play an important role in duck health and production. Here, we constructed the first relatively comprehensive duck gut microbial gene catalog (24 million genes) and 4437 metagenome‐assembled genomes using 375 GIT metagenomic samples from four different duck breeds across five intestinal segments under two distinct rearing conditions. We further characterized the intestinal region‐specific microbial taxonomy and their assigned functions, as well as the temporal development and maturation of the duck gut microbiome. Our metagenomic analysis revealed the similarity within the microbiota of the foregut and hindgut compartments, but distinctive taxonomic and functional differences between distinct intestinal segments. In addition, we found a significant shift in the microbiota composition of newly hatched ducks (3 days), followed by increased diversity and enhanced stability across growth stages (14, 42, and 70 days), indicating that the intestinal microbiota develops into a relatively mature and stable community as the host duck matures. Comparing the impact of different rearing conditions (with and without water) on duck cecal microbiota communities and functions, we found that the bacterial capacity for lipopolysaccharide biosynthesis was significantly increased in ducks that had free access to water, leading to the accumulation of pathogenic bacteria and antibiotic‐resistance genes. Taken together, our findings expand the understanding of the microbiome signatures linked to intestinal regional, temporal development, and rearing conditions in ducks, which highlight the significant impact of microbiota on poultry health and production.

## Highlights

- A catalog of microbial genes and metagenome‐assembled genomes from the duck gut microbiome.
- The microbiota within different duck intestinal segments exhibits both similarities and distinctive taxonomic and functional differences.
- Significant shifts in microbiota composition have been observed in newly hatched ducks at 3 days of age, followed by increased diversity and enhanced stability across growth stages (14, 42, and 70 days).
- Ducks with free access to water have shown an increased bacterial capacity for lipopolysaccharide biosynthesis, leading to the accumulation of pathogenic bacteria and antibiotic‐resistance genes.

## Graphical abstract

In this study, we constructed the first relatively comprehensive duck gut microbial gene catalog and metagenome‐assembled genomes using 375 duck gastrointestinal tract metagenomic samples from four different duck breeds across five intestinal segments under two distinct rearing conditions. Furthermore, the present study expands the understanding of the duck microbiome signatures linked to intestinal regional, temporal development, and rearing conditions in ducks, highlighting the significant impact of microbiota on poultry health and production.

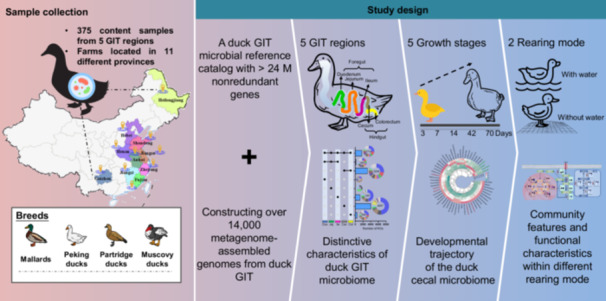

## INTRODUCTION

The poultry population in China accounts for approximately a quarter of the global poultry, and duck meat has become one of the most widely consumed animal meat [1]. The duck microbiota plays a crucial role in functions, such as nutrient digestion, immune system development, and feed efficiency improvement [2, 3, 4]. Investigations have documented alterations in duck microbial structure induced by nutrition [5], antibiotics [6], ambient temperature [2], and microplastic exposures [7], highlighting the pivotal significance of the duck microbiome in health maintenance and disease prevention.

The duck gastrointestinal tract (GIT) is a multiorgan system with great regional diversity and harbors a dynamic population of microorganisms [8]. However, previous duck gut studies have been focused more on cecal or fecal microbiota [5, 9, 10] and less attention has been given to exploring regional organization and functional potentials of the duck microbiome. Additionally, the early‐life microbiome has potential life‐long effects on host metabolism and health [11]. While recent works have revealed the temporal development and succession of chicken microbiota [12, 13], the duck gut microbiota were associated with management systems or ages only using 16S ribosomal RNA sequencing [14, 15]. Thus, a comprehensive investigation based on deep metagenomics and large‐scale sampling is required to understand the age‐associated signatures of the duck microbiome.

Antibiotic‐resistance genes (ARGs) have accelerated microbial threats to human and animal health in the last decade [16]. Furthermore, the abundance and diversity of ARGs were reported to be associated with farm environments [17] and rearing systems [18]. Duck wastes were a potential reservoir of novel ARGs [19] and further understanding of the association between the gut microbiota and ARGs across different rearing conditions would provide insights for optimizing duck management practices.

Currently, comprehensive gut microbial gene catalogs have been established for humans [20], cattle [21], mice [22], chickens [23], and pigs [24], facilitating research on gut microbiota in these host species. In this study, we obtained a nonredundant GIT microbial gene catalog (RGMGC) with 24,602,722 genes and reconstructed a total of 4437 bacterial and archaeal genomes and metagenome‐assembled genomes (MAGs) using 375 duck samples covering four different breeds (Mallard, Partridge, Peking, and Muscovy), five intestinal segments, and two rearing systems. Our metagenomic analyses revealed the region‐specific signatures and assigned functions of the duck GIT microbiome. We further tracked the developmental trajectory and maturation of the duck gut microbiome across growth stages (3, 7, 14, 42, and 70 days). Notably, we elucidated the effect of rearing conditions on modifying the duck microbiota function and antimicrobial resistance. Collectively, this work expands our current comprehension of the significant impact of microbiota on poultry health and production.

## RESULT

### Duck microbial gene catalog construction and metagenome‐assembling

**Figure 1:**
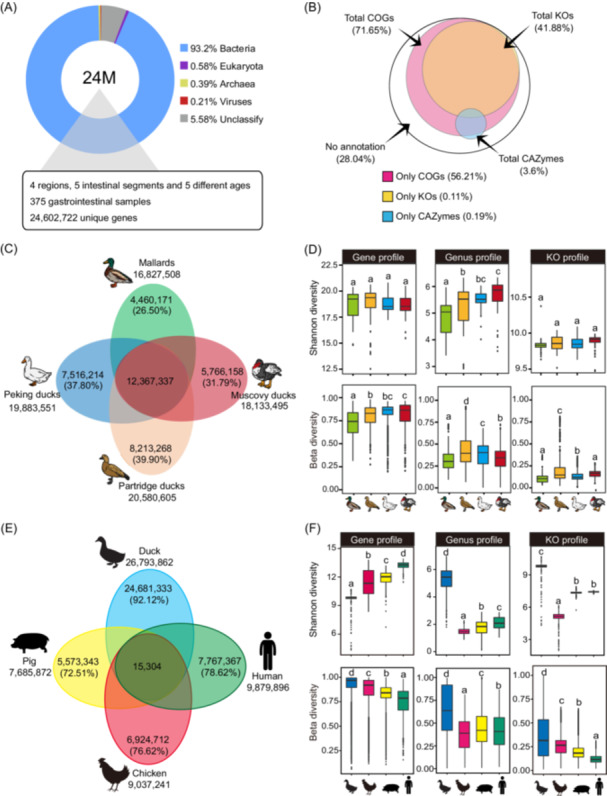
Duck gastrointestinal tract (GIT) microbial reference gene catalog. (A) Taxonomic annotations for the duck GIT microbial reference gene catalog (RGMGC) were analyzed to provide a breakdown of the taxonomic composition. (B) The RGMGC was annotated based on three functional categories (COGs, KOs, and CAZymes). Percentages of identified genes in the specified functional categories are shown. (C) Venn diagram was constructed to illustrate the distribution of unique and shared genes among the catalogs of different duck breeds. (D) Alpha (Shannon index) and beta diversities at the gene, genus, and KO function levels among four breeds. Data are shown as box plots. (E) Venn diagram was employed to illustrate the overlap and unique genes present in the catalogs of ducks, chickens, pigs, and humans. (F) Alpha and beta diversities, at the gene, genus, and KO function levels, were assessed among duck, pig, chicken, and human. Data are shown as box plots. CAZymes, carbohydrate‐active enzymes; COGs, clusters of orthologous groups; KO, KEGG orthologous.

We performed shotgun metagenomic sequencing of genomic DNA extracted from 375 samples and obtained a total of 6.8 terabytes (Tb) of Illumina sequence data (Figure S1). After clustering at 95% nucleotide sequence identity, we obtained a nonredundant GIT microbial gene catalog with 24,602,722 genes at an average length of 650 bp (Figure 1A). According to currently available databases, the genes in the RGMGC were taxonomically classified as originating from bacteria (93.2%), archaea (0.39%), eukaryotes (0.58%), and viruses (0.21%) (Figure 1A). Additionally, 71.65%, 41.88%, and 3.6% were annotated to clusters of orthologous groups of protein, KEGG orthologous groups (KOs), and carbohydrate‐active enzymes (CAZymes), respectively (Figure 1B).

We conducted a comparative analysis of the gene catalog across the four breeds, revealing distinct variations in the number of presented genes, with the order: Partridge > Peking > Muscovy > Mallard. Using the pairwise overlap analysis, we found approximately 60% shared genes, with 39.90% for Partridge, 37.80% for Peking, 31.79% for Muscovy, and 26.50% for Mallard, respectively (Figure 1C). To evaluate the microbial composition, we assessed the relative abundance of genes, genera, and KOs by employing the microbial Shannon indexes and beta diversity measurements for each breed (Figure 1D). The microbial alpha diversities, as reflected by the gene and KO profiles, were relatively consistent among the four breeds. However, we also observed a slight increase in the genus profiles from Mallard to that from Partridge, Peking, and Muscovy (Figure 1D).

We further compared the gene catalog to that of humans (*Homo sapiens*, 9.9 M), pigs (*Sus scrofa domesticus*, 7.7 M), and chickens (*Gallus gallus domesticus*, 9.04 M). Notably, the gene catalogs for these species were primarily derived from fecal samples in the case of humans and pigs, while for chickens, content samples from various intestinal compartments were utilized. By conducting pairwise overlap analyses at the gene sequence level, we observed that each species possessed a significant proportion of unique genes, with over 70% of genes being exclusive to the respective species (92.12% for ducks, 72.51% for pigs, 76.61% for chickens, and 78.62% for humans). Conversely, only a minimal percentage of genes (~0.9%) were shared among ducks, chickens, humans, and pigs (Figure 1E). Moreover, we found that alpha and beta diversities of ducks had a relatively higher variance than that of other species at both genus taxonomic and functional levels (Figure 1F).

**Figure 2:**
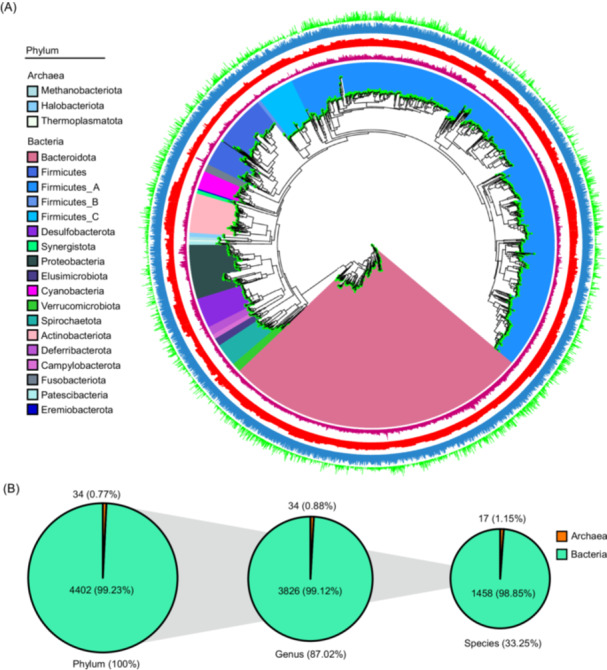
Generation and quality assessment of metagenome‐assembled genomes (MAGs) in ducks. (A) Phylogenetic tree is constructed using PhyloPhlAn, which shows the identified 4437 MAGs sampled from various regions of the duck gastrointestinal tract (GIT). Clades are colored according to phyla. Genome information is presented in the outer layers. From the inner circle to the outer circle: contamination, GC rate, completeness, and genome size. (B) The proportions and specific numbers of genome bins at the phylum, genus, and species levels.

We then investigated the distribution of dominant bacterial phyla based on the assigned MAGs (Figure 2A). The most abundant phyla were Firmicutes_A (*n* = 1920), primarily composed of classes Bacteroidia and Clostridia, followed by Bacteroidota (*n* = 1089). Additionally, Firmicutes (*n* = 310), Proteobacteria (*n* = 212), Actinobacteriota (*n* = 152), and Desulfobacterota (*n* = 119) were also prominent in the sampled duck microbiome. Regarding archaea phyla, the dominant groups were Methanobacteriota (*n* = 14), Halobacteriota (*n* = 15), and Thermoplasmatota (*n* = 5). Of the MAGs, approximately 99.23% could be annotated at the phylum level for bacteria, while 0.77% were annotated for archaea. At the genus level, 87.02% of MAGs were assigned to bacterial genera, with 99.12% falling within the bacterial domain and the remaining 0.88% being assigned to archaeal genera. Notably, a considerable portion of MAGs (33.25%) was further classified into species‐level genome bins (SGBs), encompassing 98.85% bacterial species and 1.15% archaeal species (Figure 2B).

### Distinctive taxonomic characteristics of GIT regional organization and functional potentials

**Figure 3:**
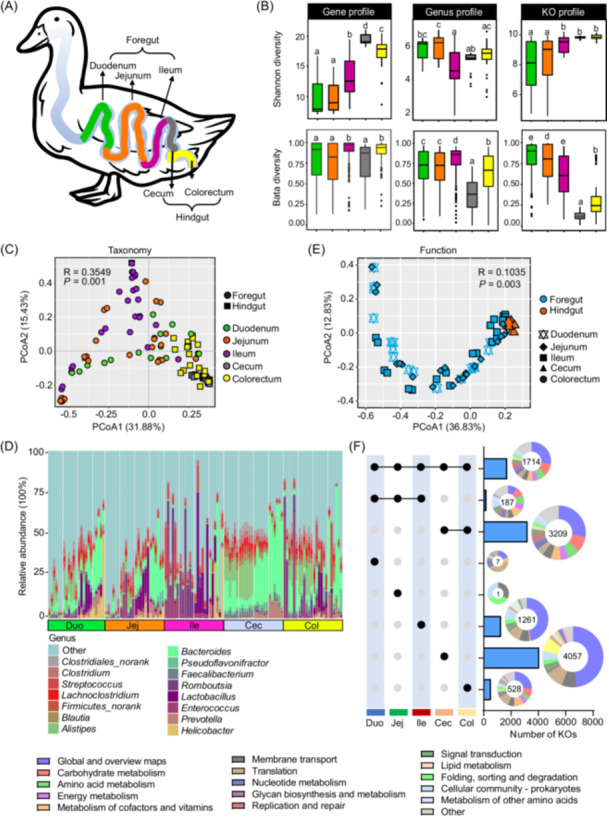
Gastrointestinal tract (GIT) functional and taxonomic variability in ducks. (A) Diagram of duck intestinal tract. The microbial densities in the foregut and hindgut were labeled. (B) Alpha (Shannon index) and beta diversities at the gene, and KO function levels among duck GIT. Data are shown as box plots. PCoA analysis based on Bray–Curtis distance of taxonomy (C) and function (D) among the GIT. (E) Relative abundances of the dominant microbial genera present within different regions of the GIT. (F) Comparison of the levels of functional KOs of the microbiome across regions of the duck GIT. The left panel shows sets included in the intersection and independent sites, and the right bar or pie charts show the categories of the KEGG pathway in these sets. The major enriched categories are shown in the legend. KO, KEGG orthologous; PCoA, principal coordinates analysis.

To investigate the distinctive characteristics of duck intestinal tract metagenomes, samples of GIT (the duodenum, jejunum, ileum of the small intestine, the cecum, and colon of the large intestine; *n* = 24) were collected (Figure S2). Distinguished by the difference in morphology and function, the duck intestinal tract can be divided into the foregut (duodenum, jejunum, and ileum) and hindgut (cecum and colorectum, Figure 3A).

Microbial diversities were largely similar within foregut compartments, showing a slight incremental trend from the duodenum to the ileum (Figure 3B). A similar pattern was observed between hindgut compartments (Figure 3B). However, microbial diversities were higher in the foregut than in the hindgut, as indicated by alpha and beta diversity measures (Figure 3B). Principal coordinates analysis (PCoA) analysis confirmed a noticeable separation between the foregut and hindgut compartments (Figure 3C). Furthermore, our investigation into the microbial taxa across the five regions of the GIT revealed striking variations. For example, Firmicutes exhibited higher abundance in the foregut compartments, whereas Bacteroidetes were more abundant in the cecum and colorectum regions (Figure S3A). Additionally, specific microbial taxa showed distinct enrichment in certain regions. For instance, *Helicobacter* spp. and *Prevotella* spp. were predominantly found in the duodenum region; *Lactobacillus* spp. and *Enterococcus* spp. were more enriched in the ileum region; *Bacteroides* spp., *Alistipes* spp., *Clostridium* spp., and *Blautia* spp. were more prevalent in the large intestine; and *Escherichia* spp. exhibited relatively high relative abundance in the small intestine (Figure 3D).

When exploring the functional distribution of intestinal regions, similar patterns were observed in PCoA analysis based on the functional profiles (Figure 3E). The comparison of functional modules, such as KOs, across the intestinal regions provided further insights into the substantial regional functional heterogeneity of the gut microbiome (Figure 3F). Specifically, the small intestine microbiome appeared to be primarily involved in cofactor, vitamin, and amino acid metabolism, while the large intestine microbiome exhibited a stronger association with lipid metabolism and functions related to protein folding, sorting, and degradation (Figure 3F).

### The regional signatures of CAZymes in the duck GIT microbiome

**Figure 4:**
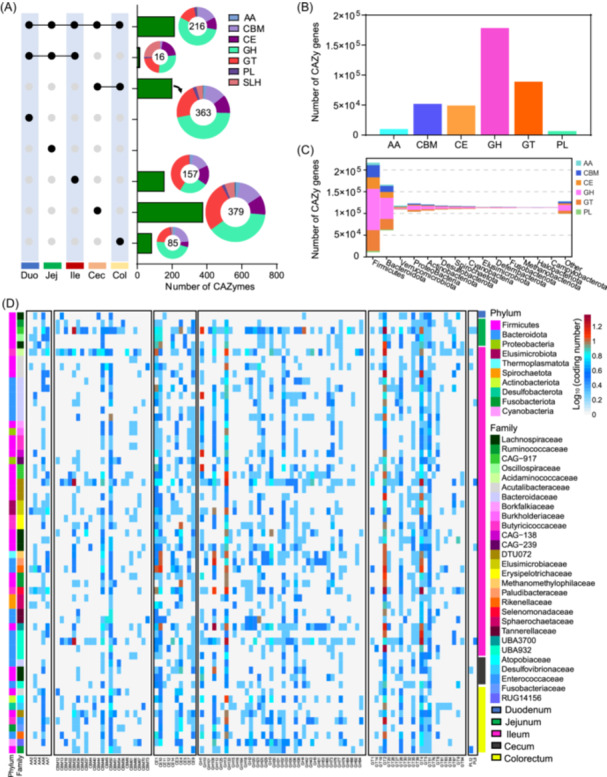
The genomic information of GIT‐associated metagenome‐assembled genomes (MAGs) that possess genes encoding carbohydrate‐active enzymes (CAZymes). (A) Comparison of the levels of CAZymes of the microbiome across regions of the duck GIT. The left panel shows sets included in the intersection and independent sites, and the right bar or pie charts show the categories of the CAZymes in these sets. The major enriched categories are shown in the legend. (B) The number of CAZymes genes based on MAGs. (C) Major phyla categorize the distribution of CAZyme families in genomes. The analysis is based on the number of families annotated in each genome. (D) The heatmap indicates the number of genes encoding CAZyme in the 60 most differentially enriched genomes identified across GIT based on the LEfSe analysis. The major MAG categories are shown in the legend. AAs, auxiliary activities; CBMs, carbohydrate‐binding modules; CE, carbohydrate esterases; GH, glycoside hydrolases; GIT, gastrointestinal tract; GT, glycosyl transferases; LDA, linear discriminant analysis; LefSe, LDA effect size; PL, polysaccharide lyases; SLH, S‐layer homologous.

CAZymes are the most important enzymes for the metabolism of complex carbohydrates in the GIT. Consistently, we found a higher abundance of carbohydrate esterases (CEs) and glycosyl transferases (GTs) in the foregut microbiome, suggesting their capacity for catalyzing substituted saccharides and sugar moiety transfers. In contrast, the larger intestines exhibited a larger proportion of glycoside hydrolases (GH), indicating their ability to hydrolyze glycosidic bonds in carbohydrates (Figure 4A). To identify CAZyme abundance associated with different segments of the intestine, we performed the linear discriminant analysis (LDA) effect size (LEfSe) analysis, and the results are presented as a heatmap in Figure S4. Notably, no CAZyme families were found to be significantly enriched in the colorectum based on the applied filter criteria (LDA > 3.5; *p* < 0.01). This is likely because the CAZyme features in the colorectum are similar to those observed in the cecum segment. Specific CAZyme families exhibited distinct abundance patterns in different intestinal segments. For instance, in the duodenum segment, CBM13/16, GH11, GH7/143/20/24/28/5/76/89, and PL27/6 were found to be more abundant. Conversely, CBM67, GH108/139/67/78, and GT30/51 showed higher enrichment in the jejunum (Figure S4). In the ileum section, CAZyme families CE4 and CE6, responsible for xylan degradation, were identified. The cecum segment presented a total of 46 CAZyme families belonging to carbohydrate‐binding modules (CBMs), CEs, GHs, GTs, and polysaccharide lyases (PLs). These results suggest that CAZyme families in the foregut appear to be more associated with the dietary composition among duck breeds, while they are more abundant and similar in the hindgut.

More than 30,000 CAZymes were encoded by 4437 MAGs, with the most prevalent classes being GHs, followed by GTs and CBMs (Figure 4B). The phyla Firmicutes and Bacteroidota, along with the Proteobacteria and Actinobacteriota, exhibited the largest and most diverse repertoire of CAZymes, respectively (Figure 4C). Firmicutes and Bacteroidota showed a high proportion of GHs, whereas Verrucomicrobiota showed a higher proportion of auxiliary activities (AAs) (Figure 4C). PCoA further showed the similarity and disparity of the CAZyme genes based on genomes in distinct intestinal segments (Figure S5). We also compared the abundance of MAGs enriched in the five digestive tract segments, and a total of 60 MAGs were identified based on the LEfSe analysis (LDA > 3; *p* < 0.05) (Figure 4D). For example, the foregut was especially rich with duodenum and jejunum‐associated MAGs mainly belonging to Lachnospiraceae, Ruminococcaceae, and Oscillospiraceae families (Figure 4D). Additionally, for the ileum section, Butyricioccaceae, Burkholdriaceae, Acutalibacteraceae, Borkfalkiaceae, and Bacteroidaceae were the most abundant MAGs taxonomic family (Figure 4D). For the hindgut, Lachnospiraceae accounted for a larger proportion in the cecum, while Atopobiaceae, Desulfovibrionaceae, Enterococcaceae, and Fusobacteriaceae were found in the colorectum tract. In the foregut tract, the microbiota was dominated by GHs, GTs, and CEs; for the hindgut, there was a higher proportion of CBMs and a decreased proportion of CEs. Moreover, AA5 was the most identified AA; and the CBM50 family for CBMs, the CE10 family for CEs, the GH13 family for GHs, and the GT2 family for GT, respectively (Figure 4D). These results indicate that similar CAZymes are encoded by different bacterial species in different intestinal segments.

### Developmental trajectory and maturation of the duck microbiome

**Figure 5:**
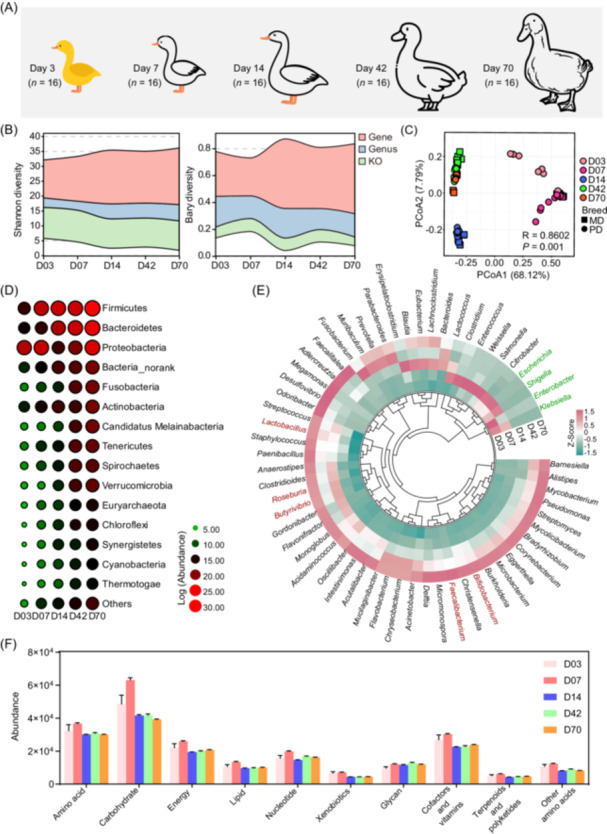
Distinctive taxonomic characteristics of GIT regional organization and functional potentials. (A) Diagram of duck growth stages. (B) Alpha (Shannon index) and beta diversities at the gene, genus, and KO function levels among the growth stages. (C) PCoA analysis based on Bray–Curtis distance of taxonomy. (D) Relative abundances of major phyla during the growth stages. (E) Relative abundances of major genera during the growth stages. (F) Comparison of KEGG functional profiles of gut microbiome during the growth stages. GIT, gastrointestinal tract; KO, KEGG orthologous; MD, Muscovy duck; PCoA, principal coordinates analysis; PD, Peking duck.

To investigate the development of gut microbial communities, we analyzed samples from ducks at five different stages (3, 7, 14, 42, and 70 days) belonging to two breeds, Muscovy ducks (MDs) and Peking ducks (PDs), two main breeds for meat production [25] (Figure 5A). For Shannon diversity, the microbial diversities were increased during duck development, peaking at day 14 for genes and genus profiles, and then remaining stable or decreasing slightly thereafter (Figure 5B). Notably, for both MD and PD, the PCoA plots displayed clear clustering of samples according to stage groups (Figure 5C). In particular, the days 42 and 70 groups exhibited a higher level of similarity, indicating a stable gut microbiota at the late stage (Figure 5C). In contrast, significant variability was revealed in the gut microbiome of new ducks (day 3), distinct from the other samples collected on 14, 42, and 70 days (Figure 5C). This observation can be attributed to the short‐term exposure to the external environment and the initial establishment of gut microbial communities during this early developmental stage. These findings indicate that the intestinal microbiota evolves into a relatively mature community as the host duck grows.

Throughout the growth stages, dominant phyla in both MD and PD breeds were Firmicutes, Proteobacteria, Bacteroidetes, Fusobacteria, and Actinobacteria. Firmicutes, the most abundant phyla, increased steadily from day 3 to day 70. While Proteobacteria declined from day 3 to day 14, followed by a relative increase until day 70 (Figure 5D). On day 3, the identified major genera included *Klebsiella*, *Lactobacillus*, *Escherichia*, and *Enterobacter* (Figure 5E). By day 7, genera such as *Clostridium*, *Enterococcus*, and *Lactococcus* were more abundant (Figure 5E). During the middle growth period, *Blautia*, *Eubacterium*, and *Bacteroides* were enriched (Figure 5E). Toward the late growth stage, *Lactobacillus*, *Roseburia*, *Butyrivibrio*, and *Bifidobacterium* showed higher abundance (Figure 5E). Notably, typical genera including *Alistipes*, *Akkermansia*, and *Butyricicoccus* increased from day 7 and remained stable until day 70, while *Escherichia* and *Lactobacillus* gradually declined over the same period (Figure S6). Stage‐associated MAGs were further identified using LEfSe (LDA > 3.5; *p* < 0.01), and the abundance is shown as a heatmap (Figure S7). Pathogens like *Klebsiella pneumoniae*, *Enterobacter hormaechei*, *Escherichia coli*, *Salmonella enterica*, and *Escherichia flexneri* were more abundant on days 3 and 7 and decreased afterward (Figure S7). Genera such as *Blautia* spp., *Butyricicoccus* spp., *Agathobaculum* spp., and *Flavonifractor* spp. were prominent on day 14. Additionally, the genera *Alistipes* and *Lachnoclostridium* increased at the late growth stage.

Microbiome function peaked on day 7 and remained stable thereafter (Figure 5F). PCoA analysis further revealed the significant clusters in growth stages associated with functions (Figure S8). Among the total 270 KEGG pathways, 234 pathways were present throughout the growth stages, based on their average relative abundance (Figure S9A). Stage‐associated bacterial function features were identified using LEfSe (Figure S9B). For example, pathways related to sulfur, glutathione, glutamine, and glyoxylate metabolism were enriched on day 3. But on day 7, there was a shift toward nutrient uptake and energy metabolism, with pathways like the phosphotransferase system and ABC transporters, indicating the increased nutrient demand. On day 14, carbohydrate metabolism pathways, especially starch and sucrose metabolism, were prominent. While on 42 and 70 days, pathways related to amino acid metabolism and fatty acid metabolism were more abundant.

### Duck gut microbiota composition and functional characteristics in different rearing conditions

**Figure 6:**
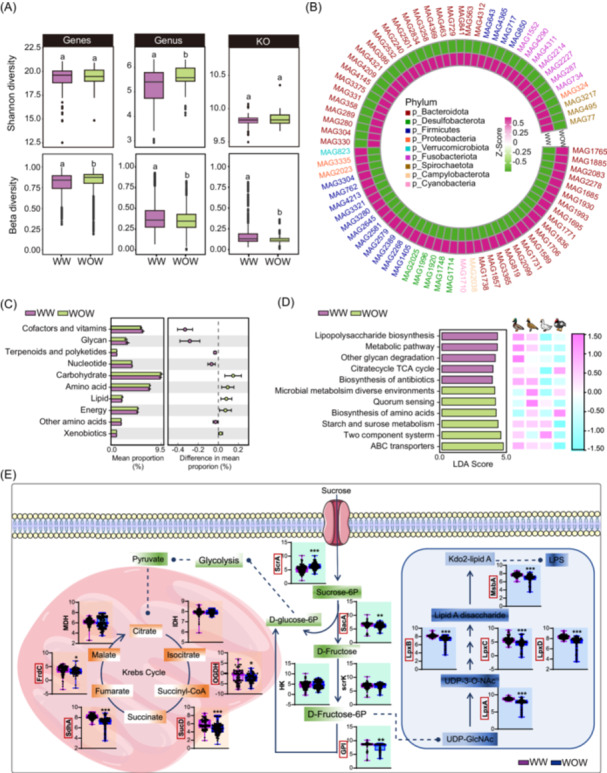
The functional alterations observed across distinct rearing conditions. (A) Alpha (Shannon index) and beta diversities at the gene, genus, and KO function levels across distinct rearing conditions. (B) The circle heatmap visualizes the enrichment of specific MAGs under different rearing conditions. The major MAG categories are shown in the legend. (C) Comparison of major KEGG functional profiles of gut microbiome under different rearing conditions. (D) LEfSe analysis of the KEGG Level 3 function for duck microbiome under different rearing conditions. (E) Comparison of KO levels involved in lipopolysaccharide biosynthesis, TCA cycle modules of the microbiome under the water and without water rearing systems. *frdC*, fumarate reductase cytochrome b subunit; *GPI*, glucose‐6 phosphate isomerase; *HK*, hexokinase; *IDH*, isocitrate dehydrogenase; KO, KEGG orthologous; LDA, linear discriminant analysis; LefSe, LDA effect size; MAG, metagenome‐assembled genome; *MDH*, malate dehydrogenase; *sacA*, the sucrase gene; *ScrA*, sucrose phosphotransferase gene; *scrK*, fructokinase; *sdhA*, succinate dehydrogenase flavoprotein subunit; *sucD*, succinate‐semialdehyde dehydrogenase; TCA, tricarboxylic acid; WOW, without water; WW, with water.

Compared with the rearing without water (WOW) condition, rearing with water (WW) increased the levels of genus and KO profiles and decreased the β‐diversity in gene profiles (Figure 6A). PCoA analysis based on the genus showed a distinct cluster between the different rearing conditions (Figure S10A). Additionally, the average relative abundance of Firmicutes and Bacteroidetes was increased and decreased, respectively, in the WOW condition (Figure S10B). MAGs were used to compare the gut microbiomes between WW and WOW rearing ducks at the species level. Annotated MAGs showed varied enrichment patterns in both groups (Figures 6B and S11). Notably, a total of 77 MAGs clustered into 36 species exhibited differential patterns. For example, among 39 MAGs that were clustered into the 18 SGB were enriched in WOW rearing ducks, including *Streptococcus alactolyticus*, *Bilophila wadsworthia*, and *Bacteroides eggerthii*, and the remaining 38 MAGs that were clustered into the 18 SGB were enriched in WW rearing ducks, mostly belonging to *Phocaeicola plebeius_A*. More importantly, we found that *Akkermansia muciniphila* were more abundant in the WOW ducks (Figures 6B and S11).

Subsequently, we compared the functional capacities of WW and WOW rearing ducks. Remarkably, the WOW condition exhibited significant enrichment in pathways related to carbohydrate, amino acid, lipid, energy, and xenobiotic metabolism. Conversely, the WW condition influenced pathways associated with cofactor and vitamin metabolism, glycan metabolism, terpenoids, and polyketide metabolism, as well as nucleotide metabolism (Figure 6C,D). Moreover, the KEGG analysis of level 1 demonstrated that the WOW rearing condition enhanced amino acid biosynthesis, starch, and sucrose metabolism, while WW ducks exhibited enriched bacterial functions involved in lipopolysaccharide biosynthesis, the tricarboxylic acid (TCA) cycle, glycan degradation, and antibiotic biosynthesis (Figure 6C,D).

An in‐depth examination of the WW‐enriched KEGG pathways led us to investigate the major enzymes involved (Figure 6E). In the WW duck microbiomes, we observed a reduction in the sucrose phosphotransferase gene (*scrA*), while the sucrase gene (*sacA*) and glucose‐6 phosphate isomerase (*GPI*) showed increased abundance. Moreover, the bacterial function associated with the TCA pathway was diminished in the WOW duck microbiome, including key enzymes such as fumarate reductase cytochrome b subunit (*frdC*), succinate dehydrogenase flavoprotein subunit (*sdhA*), and succinate‐semialdehyde dehydrogenase (*sucD*). Notably, the lpx biosynthetic cluster, which plays a crucial role in lipid A biosynthesis, exhibited enhancement under the WW duck microbiome, with increased abundance of *lpxA*, *lpxB*, *lpxC*, and *lpxD* (Figure 6F). These observed variations in bacterial functions highlight the potential metabolic adaptations of microbiomes in WW ducks.

### Antimicrobial resistance in ducks with different rearing conditions

**Figure 7:**
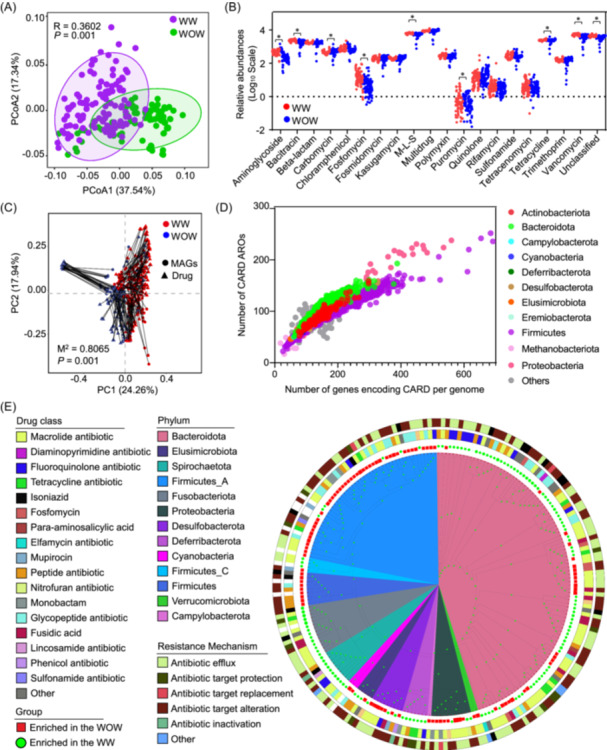
The antibiotic‐resistance genes (ARG) profiles under the different rearing conditions. (A) PCoA analysis based on Bray–Curtis distance of ARG profiles. (B) Relative abundances (log10 scale) of drug class found in each sample. Durg classes are ordered on the *x*‐axis and each dot represents a WW (red) and WOW (green) duck. The higher the vertical position of the dots on the *y*‐axis, the higher the relative abundance of the drug class. (C) Procrustes analysis showed the correlation between MAG composition and drug class levels. (D) The number of encoding CARD antibiotic resistance ontologys among the pregenome. (E) The maximum likelihood phylogenetic analysis of the microbial genomes enriched under different rearing conditions. The circle heatmap depicts the drug class and antibiotic‐resistance mechanisms. MAG, metagenome‐assembled genome; M‐L‐S, macrolide‐lincosamide‐streptogramin antibiotic; PC, principal component; PCoA, principal coordinates analysis; WOW, without water; WW, with water.

We conducted a further analysis of ARGs in ducks, with a particular focus on the distribution patterns between WOW and WW rearing conditions. Compared with WOW ducks, the gut microbiome of WW ducks had significant beta diversity and a slight increase in Shannon diversity of ARGs (Figures 7A,B and S12A). Compared with the WOW group, WW ducks had a high abundance of ARGs related to multidrug, tetracycline, beta‐lactam, carbomycin, chloramphenicol, sulfonamide, fosmidomycin, kasugamycin, and trimethoprin, but a low abundance of vancomycin, bacitracin, fusaric‐acid, macrolide‐lincosamide‐streptogramin antibiotic, aminoglycoside, puromycin, and fosfomycin (Figure S12B). For ARG subtypes, *bla*1, *van*Z, *erm*B, *rmt*F, *fos*X, and *tet*V were significantly downregulated in the WOW group, and *mex*B was increased in the WW ducks (Figure S12C).

Procrustes analysis revealed a significant correlation between bacterial communities and ARG compositions (Figure 7C). Genome‐based ARG profiles were further analyzed and showed that major phyla Actinobacteria, Bacteroidota, Firmicutes, and Proteobacteria were mainly responsible for the comprehensive antibiotic resistance database (CARD) encoding (Figure 7D). The phylogenetic tree, illustrating 227 MAGs enriched in both WOW and WW ducks, showed that MAGs enriched in the WW ducks mostly belonged to the phyla Bacterioidota and Proteobacteria, which largely encoded Fluoropyrimidine, macrolide, glycopeptide, and tetracycline antibiotics (Figure 7E). Furthermore, resistance mechanisms were divided into five categories, including antibiotic efflux, target protection, target replacement, target alteration, and inactivation (Figure 7E).

## DISCUSSION

### Regional signatures of the duck GIT microbiome

The GIT is a complex system with regional diversity, hosting a vast array of gut microbes and serving diverse functions pivotal to poultry production and health [26, 27, 28, 29]. We found a different trend in gut microbiome diversity along the digestive tract, as alpha diversity was highest in the small intestine but nearly identical in the cecum and colon. The dominance of lactic acid‐producing bacteria in the small intestine, including *Helicobacter* spp., *Enterococcus* spp., and *Lactobacillus* spp. aligns with their known roles in fermenting carbohydrates and promoting gut health [30, 31]. Their prevalence suggests an active fermentation process that contributes to the production of short‐chain fatty acids (SCFAs), which serve as an energy source for the host and play a role in immune modulation [32, 33].

In contrast, the predominance of mucin‐degrading taxa such as *Bacteroides* spp. and *Alistipes* spp. in the large intestine highlights the importance of mucin degradation and utilization of complex carbohydrates in this gut region [34, 35]. Additionally, the higher abundance of *Faecalibacterium* and *Blautia* in the cecum section of MD and PD suggested their involvement in fiber degradation and SCFA production [36, 37]. Notably, *Faecalibacterium*, has been associated with butyrate production, which has anti‐inflammatory properties and supports intestinal epithelial integrity [38, 39]. The presence of CAZyme families in *Faecalibacterium* indicates their abilities to utilize complex carbohydrates, such as pectin and glycans [40, 41], further highlighting their roles in fiber metabolism.

CAZymes such as cellulase, hemicellulases, and oligosaccharide‐degrading enzymes, encoded by gut microbiota, collectively play important roles in carbohydrate utilization in the GIT [42, 43]. Our study revealed a rich repertoire of CAZyme genes in the duck microbiome, encompassing a wide range of enzyme families, which likely facilitates the efficient utilization of various carbohydrate substrates throughout the GIT, supporting the metabolic requirement of both the gut microbes and the host. Notably, CAZyme families in the foregut appeared to be more associated with the dietary composition, compared with those in the hindgut. We suspect that the hindgut, which comes later in the digestive process, may exhibit more stable CAZyme abundance and similarity due to its role in fermentation and absorption, which may not be directly impacted by breeds or specific dietary preferences [44]. Furthermore, glycosidic linkage hydrolysis‐related CAZyme families, such as GHs and PLs, were found to be more abundant in the hindgut microbiome compared with those in the foregut. These spatial variations in CAZyme composition and the predominance of specific enzyme families highlight the importance of regional specialization in carbohydrate utilization, contributing to our understanding of the diverse metabolic capabilities of the duck microbiome.

### Developmental trajectory of the duck cecal microbiome

The progressive maturation of gut microbiota observed in various animal species, including pigs [45], humans [46], chickens [13], and calves [47], reflects the dynamic process of microbial colonization during different life stages. A previous study revealed the variability in the gut microbiome of newly hatched chicks compared with elder samples [12]. Consistent with this, our findings revealed notable shifts in the microbiota composition within the first 7 days, followed by increased diversity and enhanced stability at late stages. This pattern closely resembles the changes observed in the early‐life stages of human gut microbiota, suggesting similar microbial dynamics across species [48]. PCoA analysis displayed that the cecal microbiota community exhibited clustering based on age rather than duck breeds, indicating a successional development process. These findings collectively suggest that the intestinal microbiota in ducks evolves into a relatively mature and stable community as the host duck develops.

A previous study on Pekin ducks emphasized the prevalence of Proteobacteria during early cecal microbiome development [8]. Consistently, our study detected a higher abundance of Proteobacteria on day 3. The enrichment of Proteobacteria, including known pathogenic species such as *K. pneumoniae*, *E. coli*, and *E. flexner* were more abundant in the early‐life stages, reflecting the short‐term exposure to the environment and the initiation of gut microbial communities. Conversely, the increase of *Agathobaculum*, a strict anaerobic butyrate‐producing gut bacterium during the duck middle growth stages, indicated a shift toward a more stable, healthy, and mature microbial community [49]. Additionally, other key butyrate‐producing bacteria, such as *Blautia* and *Butyricicoccus*, known for their probiotic characteristics, were consistently observed during middle growing stages, similar to the previous research [50]. The presence of these beneficial species suggested a transition of the gut environment toward a healthier stage, which may contribute to improved growth performance and disease resistance in ducks.

The enrichment of specific metabolic pathways at different stages of duck growth reflected the dynamic interaction between the developmental requirement of the host and the metabolic capabilities of microbial communities. For example, on day 3, the prominence of sulfur metabolism, along with glutathione and glutamine metabolism, suggested an early emphasis on antioxidant defense and amino acid metabolism in ducks [51]. By days 7–14, there was a shift toward nutrient uptake and energy metabolism, as evidenced by the presence of pathways related to carbohydrate metabolism, indicating an increased demand for nutrient absorption and utilization to support the growing metabolic requirement in the developing duck [52]. In the late stage of 42–70 days, there was a shift toward pathways involved in amino acid and fatty acid metabolism, which reflected the increasing demand for protein synthesis and adipose tissue development [53]. Further research into the specific mechanisms underlying these metabolic changes could potentially provide targeted interventions to enhance duck production efficiency.

### Duck cecal microbiome changes under different rearing systems

Previous studies have investigated the impacts of different housing systems on host performance traits [54], antioxidant defense [55], as well as microbial diversity [56], highlighting the importance of environmental factors in the poultry industry. In recent years, to meet the increasing demand for duck meat and improve the efficiency of poultry meat output, the duck industry has gradually moved toward intensive and large‐scale development in China. Cage systems with a large feeding capacity and high space utilization have become a trend. We thus divided the ducks into WOW and WW groups. “WOW” has cage systems featuring a plastic mesh floor without access to water, while “WW” signifies ducks with free access to water for swimming.

Our study revealed a higher alpha diversity of the gut microbiome at the genus level in ducks reared WOW compared with those reared WW. This suggests that the absence of water access may lead to alterations in gut microbial community structure and diversity. Specifically, the gut microbiome of WOW ducks exhibited a significantly higher abundance of *Bacteroides* spp., including *Bacteroides cellulosilyticus*, *B. eggerthii*, *Bacteroides uniformis*, and *Bacteroides xylanisolvens*. These species are known to possess the ability to utilize both dietary and endogenous glycans, contributing to the production of beneficial end products such as SCFAs, which are primary metabolites of host and microbial metabolism [57]. Additionally, we observed a significant enrichment of the bacterial species *A. muciniphila* in the gut microbiome of WOW ducks. *A. muciniphila* plays a crucial role in maintaining the integrity of the intestinal barrier and modulating the host immune response [58]. These findings suggest potential adaptations of duck gut microbial communities to water‐restricted environments.

On the other hand, species from *Paraprevotella* spp., and *Phocaeicola* spp. were enriched in the WW ducks. *Paraprevotella* spp. is characterized by the production of succinic and acetic acid as major fermentation products [59]. *Phocaeicola* spp. are known to be highly effective producers of succinate, acetate, and propionate [60]. Conversely, the enrichment of *Fusobacterium* spp. in WW ducks raises concerns about their association with serious infections, particularly in clinical settings [61]. For example, *Fusobacterium necrophoru* is known to cause infections, especially in younger individuals [62]. Thus, the observed differences in microbial composition between WOW and WW ducks indicate potential adaptations and interactions between the gut microbiota and rearing conditions and highlight the complex interplay between environmental factors, host physiology, and gut microbial communities.

### Different rearing conditions modulated bacterial function and ARGs

Excessive LPS exposure can trigger inflammatory responses and contribute to gut barrier dysfunction, leading to increased susceptibility to infections and inflammatory disorders [63]. The elevated expression of key enzymes involved in lipid‐A‐disaccharide synthesis, such as *lpxB*, *lpxC*, and *lpxD* in the gut microbiota of WW ducks, suggests an increased capacity for LPS production [64]. These findings suggest that the WW duck microbiome possesses a regulatory mechanism for LPS biosynthesis, likely through the coordinated regulation of *lpx* gene cluster enzymes. Furthermore, our findings revealed enhanced activity of the TCA cycle in the gut microbiomes of WW ducks, specifically from glycolysis. This metabolic adaptation reflects the need for efficient energy utilization to support the growth and metabolic demands of gut microbes in response to environmental cues, such as water availability.

The impact of rearing conditions on the profiles of ARGs in livestock has been well‐documented in various species [65, 66, 67]. Our metagenomic sequencing results revealed a slight enhancement in the Shannon index of ARGs in WW ducks, suggesting that the water access may have influenced the abundance and diversity of ARGs in the microbiome. Outdoor production systems offer ducks access to natural environments and environmental reservoirs of antibiotic‐resistant bacteria that may increase the likelihood of ARG transmission to poultry populations. Furthermore, we observed an enrichment of major antibiotic drug categories, including multidrug, tetracycline, and beta‐lactam resistance in the gut microbiome of WW ducks. It is recognized that aquatic environments are one of the key reservoirs and transmission routes for the spread of antimicrobial resistance in livestock populations [68]. Ducks in the WW group may accumulate and retain certain antibiotic drugs in their bodies when exposed to these drugs through water sources. These findings contribute to our understanding of the relationship between rearing conditions, gut microbiota function, and the presence of ARGs in ducks. Further research is warranted to elucidate the underlying mechanisms and consequences of these findings, which may contribute to optimizing the rearing practices and health management strategies in the poultry industry.

## CONCLUSION

In the present study, we subjected the GIT microbiomes of four duck breeds to large‐scale metagenomic sequencing and generated a relatively comprehensive reference gene catalog covering more than 24 million genes. We assembled 4437 MAGs assigned to bacterial and archaeal lineages. Our metagenomic results highlighted both the similarities and distinct taxonomic and functional differences across the duck GIT microbiota. The intestinal microbiota develops into a relatively mature community and reaches the maximum metabolic capacity during 42 days. Importantly, we identified several differences in species‐level MAGs between the WW and WOW duck gut microbiome, shedding light on the higher diversity of ARGs and increased capacity for lipopolysaccharide biosynthesis under water‐rearing conditions. These findings contribute valuable resources that offer insights into the composition and dynamics of the duck gut microbiome, serving as a foundation for future metagenomic sequencing‐based studies in the field.

## METHODS

### Experimental design and sample collection

For the metagenomic sequencing analysis, a total of 375 gastrointestinal samples were collected (Figures S1 and S2, and Table S1), which were raised on different farms located in different provinces, including Heilongjiang, Zhejiang, Jiangxi, Henan, Shanghai, Guizhou, Shandong, Jiangsu, Anhui, Fujian, and Hebei in China. The study design was divided into the four following parts (Figure S2):

(1) A total of 375 gastrointestinal samples, including four breeds (Mallard, Partridge, Peking, and Muscovy), five intestinal segments (the duodenum, jejunum, and ileum of the small intestine; and the cecum and colon of the large intestine), and five different growth ages (3, 7, 14, 42, and 70 days) were used for the construction of the duck GIT microbial gene category (Figures S1 and S2, and Table S1).
(2) To investigate the distinctive characteristics of duck intestinal tract metagenomes (*n* = 120 total samples), samples of GIT (the duodenum, jejunum, and ileum of the small intestine; and the cecum and colon of the large intestine) for the two breeds: PD (*n* = 12/intestinal segment) and MD (*n* = 12/intestinal segment) were used (Figure S2 and Table S2).
(3) To investigate the development of gut microbial communities, cecum samples of five different duck ages (3, 7, 14, 42, and 70 days) were analyzed for two breeds: PD (*n* = 8/age) and MD (*n* = 8/age) (Figure S2 and Table S3).
(4) To explore the impact of different rearing conditions on duck microbiome profiles, cecum samples from the cecum of the four breeds: Mallard (*n* = 30), Partridge (*n* = 62), Peking (*n* = 51), and Muscovy (*n* = 62) were divided into two groups: WW (*n* = 91) and WOW (*n* = 114) (Figure S2 and Table S4).

The experimental ducks raised on the farm of Zhejiang Academy of Agriculture Sciences were provided standard commercial feed according to a previous study, which mainly contained 56.5% corn, 20% soybean meal, 18% wheat, 1.85% soybean oil, 1.16% sodium carbonate, 0.64% dicalcium phosphate, and other related amino acids [8]. All other province ducks were fed with commercial formula feed satisfying the standard duck nutritional requirements. All freshly collected samples were immediately frozen in liquid nitrogen and transported to the laboratory using dry‐ice packs. Subsequently, they were stored at −80°C until total DNA extraction could be performed.

### DNA extraction and sequencing

Bacterial DNA was isolated using the QIAamp DNA Stool Mini Kit (QIAGEN) according to the manufacturer's instructions. The DNA integrity was determined by electrophoresis in 0.8% agarose gels, and the concentration and quality were determined using a Nanodrop ND‐1000 (Thermo Scientific). A metagenomic library with an insert size of 350 bp was constructed from high‐quality DNA extracted from each sample using the TruSeq DNA PCR‐Free Library Preparation Kit (Illumina) following the manufacturer's instructions and then sequenced on an Illumina NovaSeq platform according to the previous study [69].

### Construction of the duck microbial gene catalog

The sequencing generated a total of 6.8 Tb of Illumina data from the 375 samples and approximately 45.7 billion sequencing reads with a read length of 150 bp (Table S5). Adapters from the Illumina data were first trimmed using Trimmomatic [70] (v.0.33). Then, to decrease potential DNA contamination from the environment, we mapped the sequence data to host and human genomes using burrows‐wheeler aligner (BWA)‐MEM [71] (v.0.7.17). A total of 1.7 Tb of sequencing reads remained, referred to as high‐quality reads, including 338.7 GB for Mallard, 330.4 GB for Muscovy, 322.5 GB for Partridge, 313.6 GB for PDs; 15.5 GB for 3 days, 48.1 GB for 7 days, 44.5 GB for 14 days, 69.6 GB for 43 days, and 59.1 GB for 70 days (Table S5).

### De novo metagenome assembly, gene prediction, and annotation

Raw sequence reads underwent quality trimming using Trimmomatic to remove adapter contaminants and low‐quality reads [70]. Reads through quality control were then mapped against the genome by the BWA mem algorithm (parameters: −*M* −*k* 32 −*t* 16, http://bio-bwa.sourceforge.net/bwa.shtml). The reads removing duck‐genome contaminations and low‐quality data were called as clean reads and used for further analysis. The data after quality control of each sample were assembled by MegaHit (parameter: ‐min‐contig‐len 500) software to obtain contigs. A total of 60.6 million contigs were obtained with an average length of 23.4 billion sequencing (Table S6). All open reading frames were generated to a set of unique genes after clustering using cluster database at high identity with tolerance (CD‐HIT) (parameters: −*n* 9 −*c* 0.95 −*G* 0 −*M* 0 −*d* 0 −*aS* 0.9 −*r* 1) [72]. The longest sequence of each cluster (Cluster) is considered the representative sequence of each gene in the unique gene set. To calculate the gene abundance in the total sample, the salmon software [73] was used to obtain the number of reads for each gene. Unique gene sets were searched in the KEGG database using BLASTX to identify the functions of the annotated protein sequences. On the basis of the KO results of all samples, pathways were mapped through the KEGG database using annotated genes, and specific function and pathway maps for each sample were obtained. The predicted genes were converted into amino acid sequences, and the CAZy database [74] and eggNOG database [75] were annotated using DIAMOND software [76].

### Metagenomic binning

Metagenome binning was performed on each sample contigs. Briefly, high‐quality contigs were binned into MAGs using MetaBAT2 [77], MaxBin [78], and CONCOCT [79] (Figure S1). To improve the MAG assembly quality, the MetaSPAdes [80] software was used for MAG reassembly, and the data was extracted from clean data by the BWA‐MEM method. After quality control and data filtration, 4437 MAGs exceeding medium quality (completeness ≥50% and contamination ≤10%) remained (Table S7). Then apply the metaWRAP quant_bins module [81] to calculate the abundance of each MAG with default parameters based on the TPM calculation process. The estimated genome size was corrected based on the completeness and contamination following the algorithm from Nayfach et al. [82]. All genes in the bins were converted to protein sequences, resulting in the proteome for each bin. These proteomes were used for later PhyloPhlAn reconstruction of the dendrogram [81] and visualized using Evolview (v.3) [83] and iTol (v.4.3.1) [84]. dRep was applied to cluster MAGs under an ANI > 95% and 99%, and the final 1208 representative MAGs with the highest QualityScore values (defined as completeness − 5× contamination) were selected (Table S8). All genomes were annotated using GTDB‐Tk (v.0.1.6) based on the Genome Taxonomy Database [85].

### Statistical analysis

Shannon index was performed to measure the diversity of genus, genes, and KOs. The overall differences in the bacterial community structures and function profiles were evaluated based on Bray–Curtis dissimilarity values, and the differences between groups were assessed using the PERMANOVA and ANOSIM tests and then visualized using a PCoA plot. The LEfSe analysis was conducted to identify significant differences among features (genus, MAGs, functions). Statistical analysis of metagenomic profiles analysis was used to compare the differences in the function of microbial communities from different rearing conditions. The heatmap figure was constructed using the TBtools [86]. Bioinformatic analysis was performed using the OmicStudio tools at https://www.omicstudio.cn/tool [87].

## AUTHOR CONTRIBUTIONS

Lingyan Ma and Wentao Lyu performed the experiments, analyzed the data, prepared the figures, and wrote the manuscript. Wen Wang, Tao Zeng, and Qu Chen collected the samples. Jiangchao Zhao and Guolong Zhang participated in the experiments. Hua Yang, Lizhi, Lu, and Yingping Xiao contributed to the study concept and design, and revised the manuscript. All authors have read the final manuscript and approved it for publication.

## CONFLICT OF INTEREST STATEMENT

The authors declare no conflict of interest.

## ETHICS STATEMENT

The ethics application (2019ZAASLA95) was approved by the Institutional Animal Care and Use Committee of the Zhejiang Academy of Agricultural Sciences.

## Supporting information


**Figure S1**. Schematic diagram of sample collection.
**Figure S2**. Schematic representation of the collected sample and metagenomic analysis.
**Figure S3**. The Gastrointestinal Tract (GIT) phylum abundance in ducks.
**Figure S4**. Heatmap analysis showed the CAZymes enriched in the distinct intestine sections identified by LEfSe (LDA > 3.5; *p* < 0.01).
**Figure S5**. PCoA showed the similarity and disparity of the CAZyme gene coding numbers based on genomes in distinct intestine segments.
**Figure S6**. The abundance of *Alistipes*, *Akkermansia*, *Faecalibacterium*, *Butyricoccus*, *Escherichia*, and *Lactobacillus* across growth stages.
**Figure S7**. Heatmap showing stage‐associated metagenome‐assembled genomes (MAGs) identified by linear discriminant analysis (LDA) effect size (LEfSe) (LDA > 3.5; *p* < 0.01) for the Peking duck (PD) and Muscovy duck (MD).
**Figure S8**. PCoA analysis based on Bray–Curtis distance of function profiles across growth stages (day 3, 7, 14, 42, and 70).
**Figure S9**. The microbiome function alteration from ducks at different ages (day 3, 7, 14, 42, and 70).
**Figure S10**. Duck cecal microbiome changes under different rearing systems.
**Figure S11**. The species‐level genome bins (SGBs) containing metagenome‐assembled genomes (MAGs) showing different directions of enrichment in the WW and WOW groups.
**Figure S12**. The antibiotic resistance genes (ARG) profiles under different rearing conditions.

**Table S1**. Overview information of 375 samples.
**Table S2**. Samples for the GIT regional analysis.
**Table S3**. Samples for the developmental maturation of duck microbiome.
**Table S4**. Samples for the duck microbiome analysis under different rearing conditions.
**Table S5**. The information on microbial gene catalogs.
**Table S6**. Duck metagenome assembling and gene prediction.
**Table S7**. The quality of MAGs.
**Table S8**. The information on dRep MAGs.

## Data Availability

The data that support the findings of this study are openly available in NCBI at https://www.ncbi.nlm.nih.gov/bioproject/?term=PRJNA1064443, reference number PRJNA1064443 and PRJNA871932. Metagenomic sequencing data were submitted to the NCBI Sequence Read Archive (SRA) database under the study accession numbers PRJNA1064443 (https://www.ncbi.nlm.nih.gov/bioproject/?term=PRJNA1064443) and PRJNA871932 (https://www.ncbi.nlm.nih.gov/bioproject/?term=PRJNA871932). The data used are saved in GitHub (https://github.com/Lingyma/Maly2024-iMETA). Supplementary materials (figures, tables, scripts, graphical abstract, slides, videos, Chinese translated version, and update materials) may be found in the online DOI or iMeta Science http://www.imeta.science/.
