# Supplementary material for: Duck gut metagenome reveals the microbiome signatures linked to intestinal regional, temporal development, and rearing condition
Source: Imeta. 2024 May 14;3(4):e198. doi: 10.1002/imt2.198 (PMC11316934; doi:10.1002/imt2.198)
Supplement: Supplementary file 1 — Figure S1. Schematic diagram of sample collection. Figure S2. Schematic representation of the collected sample and metagenomic analysis. Figure S3. The Gastrointestinal Tract (GIT) phylum abundance in ducks. Figure S4. Heatmap analysis showed the CAZymes enriched in the distinct intestine sections identified by LEfSe (LDA > 3.5; p < 0.01). Figure S5. PCoA showed the similarity and disparity of the CAZyme gene coding numbers based on genomes in distinct intestine segments. Figure S6. The abundance of Alistipes, Akkermansia, Faecalibacterium, Butyricoccus, Escherichia, and Lactobacillus across growth stages. Figure S7. Heatmap showing stage‐associated metagenome‐assembled genomes (MAGs) identified by linear discriminant analysis (LDA) effect size (LEfSe) (LDA > 3.5; p < 0.01) for the Peking duck (PD) and Muscovy duck (MD). Figure S8. PCoA analysis based on Bray–Curtis distance of function profiles across growth stages (day 3, 7, 14, 42, and 70). Figure S9. The microbiome function alteration from ducks at different ages (day 3, 7, 14, 42, and 70). Figure S10. Duck cecal microbiome changes under different rearing systems. Figure S11. The species‐level genome bins (SGBs) containing metagenome‐assembled genomes (MAGs) showing different directions of enrichment in the WW and WOW groups. Figure S12. The antibiotic resistance genes (ARG) profiles under different rearing conditions. [file IMT2-3-e198-s001.docx]

**Support information for**

**Duck Gut Metagenome Reveals the Microbiome Signatures Linked to Intestinal Regional, Temporal Development, and Rearing Condition**

**Running title：**Gut Metagenome Reveals the Duck Microbiome Signatures

Lingyan Ma^1#^, Wentao Lyu^1#^, Tao Zeng^2^, Wen Wang^1^, Qu Chen^1^, Jiangchao Zhao^3^, Guolong Zhang^4^, Lizhi Lu^2*^, Hua Yang^1*^, Yingping Xiao^1*^

^1^State Key Laboratory for Managing Biotic and Chemical Threats to the Quality and Safety of Agro-products, Institute of Agro-product Safety and Nutrition, Zhejiang Academy of Agricultural Sciences, Hangzhou, 310021, China

^2^Institute of Animal Husbandry and Veterinary Medicine, Zhejiang Academy of Agricultural Sciences, Hangzhou, 310021, China

^3^Department of Animal Science, Division of Agriculture, University of Arkansas, Fayetteville, AR 72701, USA

^4^Department of Animal and Food Sciences, Oklahoma State University, Stillwater, OK 74078, USA

^#^These authors contributed equally: Lingyan Ma, Wentao Lyu

^*^Correspondence: xiaoyp@zaas.ac.cn (Yingping Xiao); [lulizhibox@163.com](mailto:lulizhibox@163.com) (Lizhi Lu); yanghua@zaas.ac.cn (Hua Yang)

**Supplementary Figures**

**
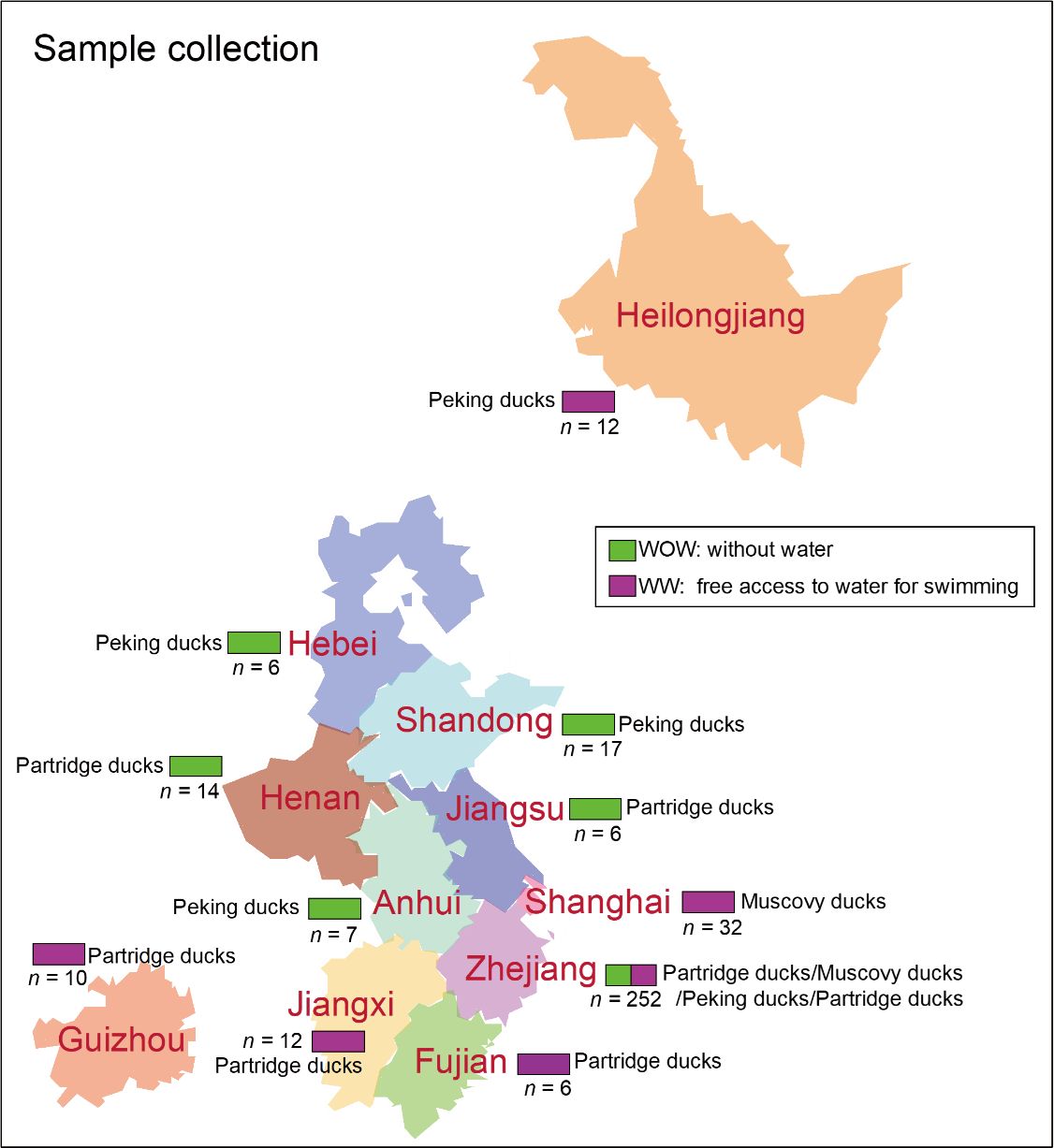
**

**Figure S1** Schematic diagram of sample collection. For the metagenomic sequencing analysis, a total of 375 gastrointestinal samples, which were raised in different farms located in different provinces, including Heilongjiang, Zhejiang, Jiangxi, Henan, Shanghai, Guizhou, Shandong, Jiangsu, Anhui, Henan, Fujian, Hebei in China were collected.


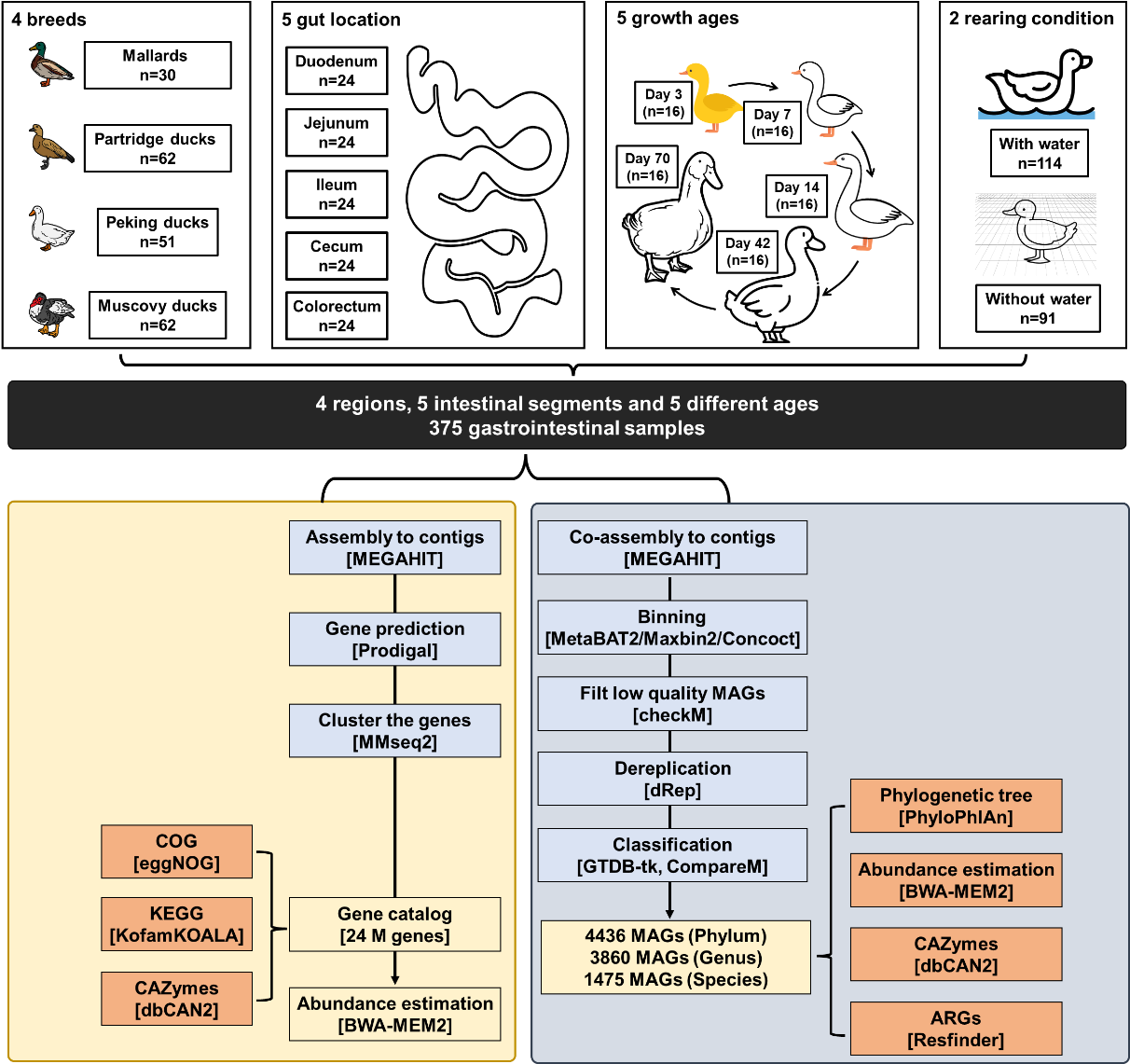


**Figure S2** Schematic representation of the collected sample and metagenomic analysis. Firstly, 375 gastrointestinal samples, including 4 breeds, 5 intestinal segments, and 5 different growth ages were used for the construction of the duck GIT microbial gene category. Secondary, to investigate the distinctive characteristics of duck intestinal tract metagenomes, samples of GIT for the two breeds: Peking duck (PD, *n* = 12/intestinal segment) and Muscovy duck (MD, *n* = 12/intestinal segment); To investigate the development of gut microbial communities, cecum samples of five different duck ages were analyzed for two breeds, Peking duck (PD, *n* = 8/age) and Muscovy duck (MD, *n* = 8/age); To explore the impact of different rearing conditions on duck microbiome profiles, cecum samples from the cecum of the four breeds: Mallards (n = 30), Partridge duck (*n* = 62), Peking duck (*n* = 51) and Muscovy duck (*n* = 62) were divided into the two group: with water (WW, *n* = 91), without water (WOW, *n* = 114).


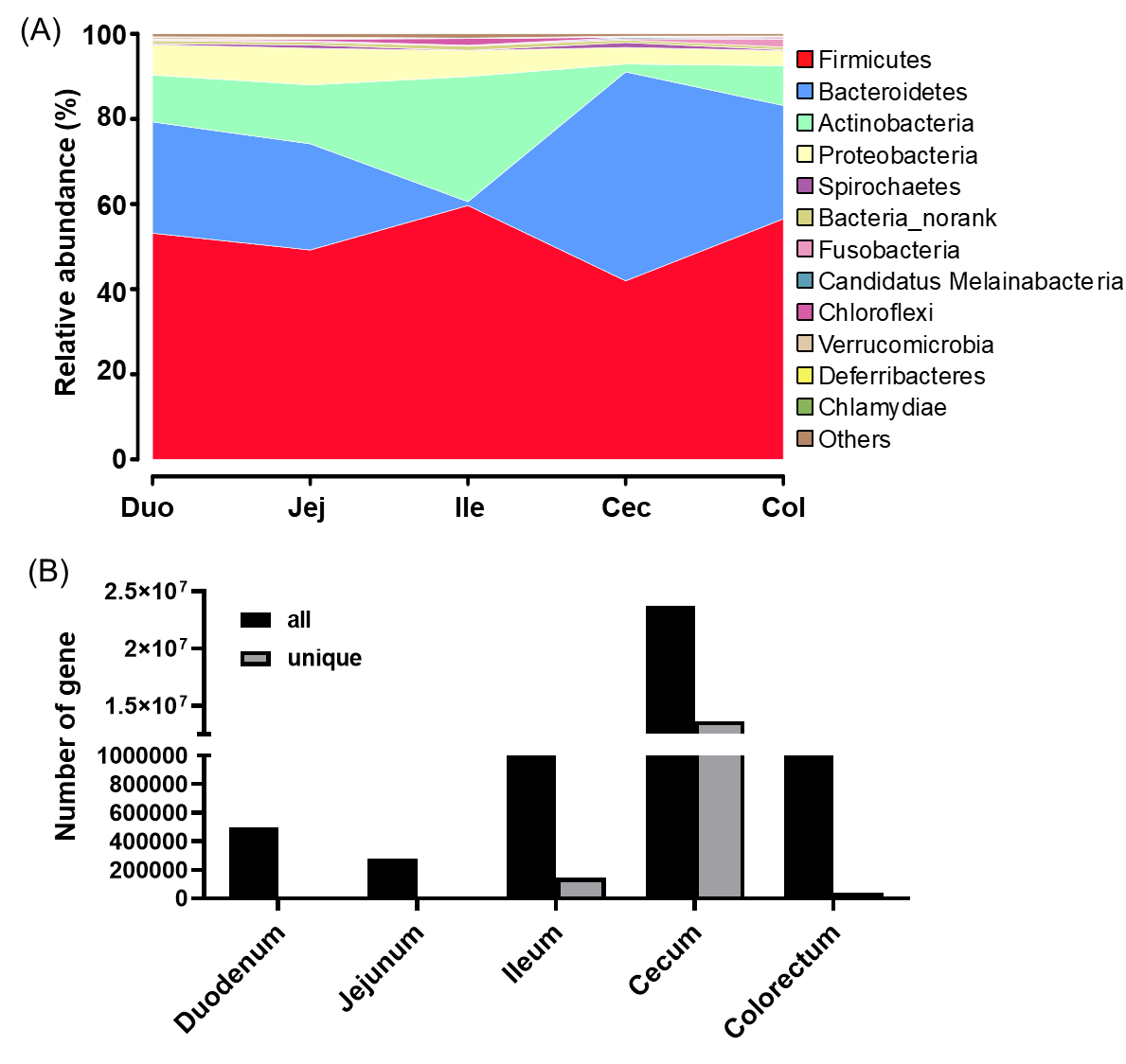


**Figure S3** The Gastrointestinal Tract (GIT) phylum abundance in ducks. (A) Relative abundance of major phyla across the GIT in ducks. (B) Number of the total and unique genes encoded among the GIT sections. Duo: Duodenum; Jej: Jejunum; Ile: Ileum; Cec: Cecum; Col: Colorectum.


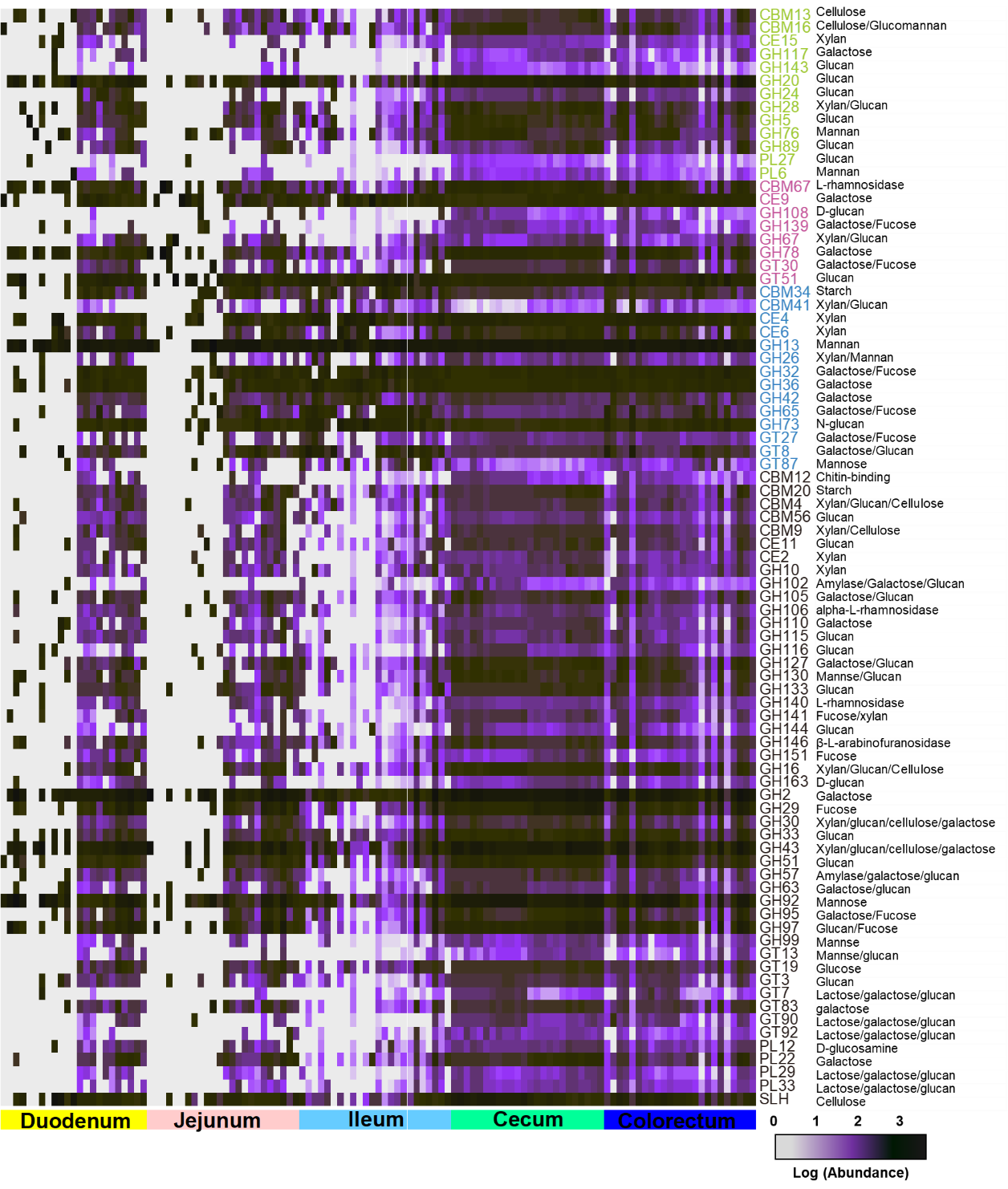


**Figure S4** Heatmap analysis showed the CAZymes enriched in the distinct intestine sections identified by LEfSe (LDA > 3.5; *p* < 0.01). Heatmap shows the average relative abundances on a log scale.


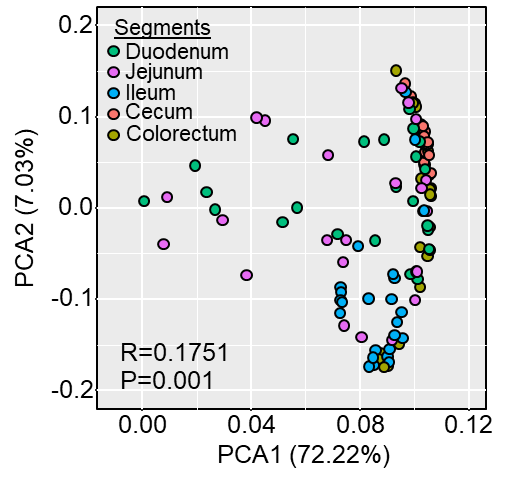


**Figure S5** PCoA showed the similarity and disparity of the CAZyme gene coding numbers based on genomes in distinct intestine segments.


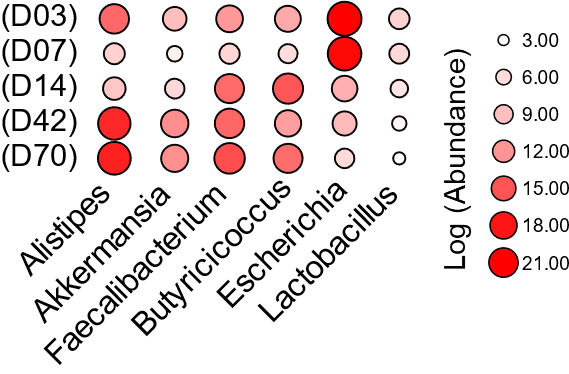


**Figure S6** The abundance of *Alistipes*, *Akkermansia*, *Faecalibacterium*, *Butyricoccus*, *Escherchia* and *Lactobacillus* across growth stages. Heatmap shows the average relative abundances on a log scale.


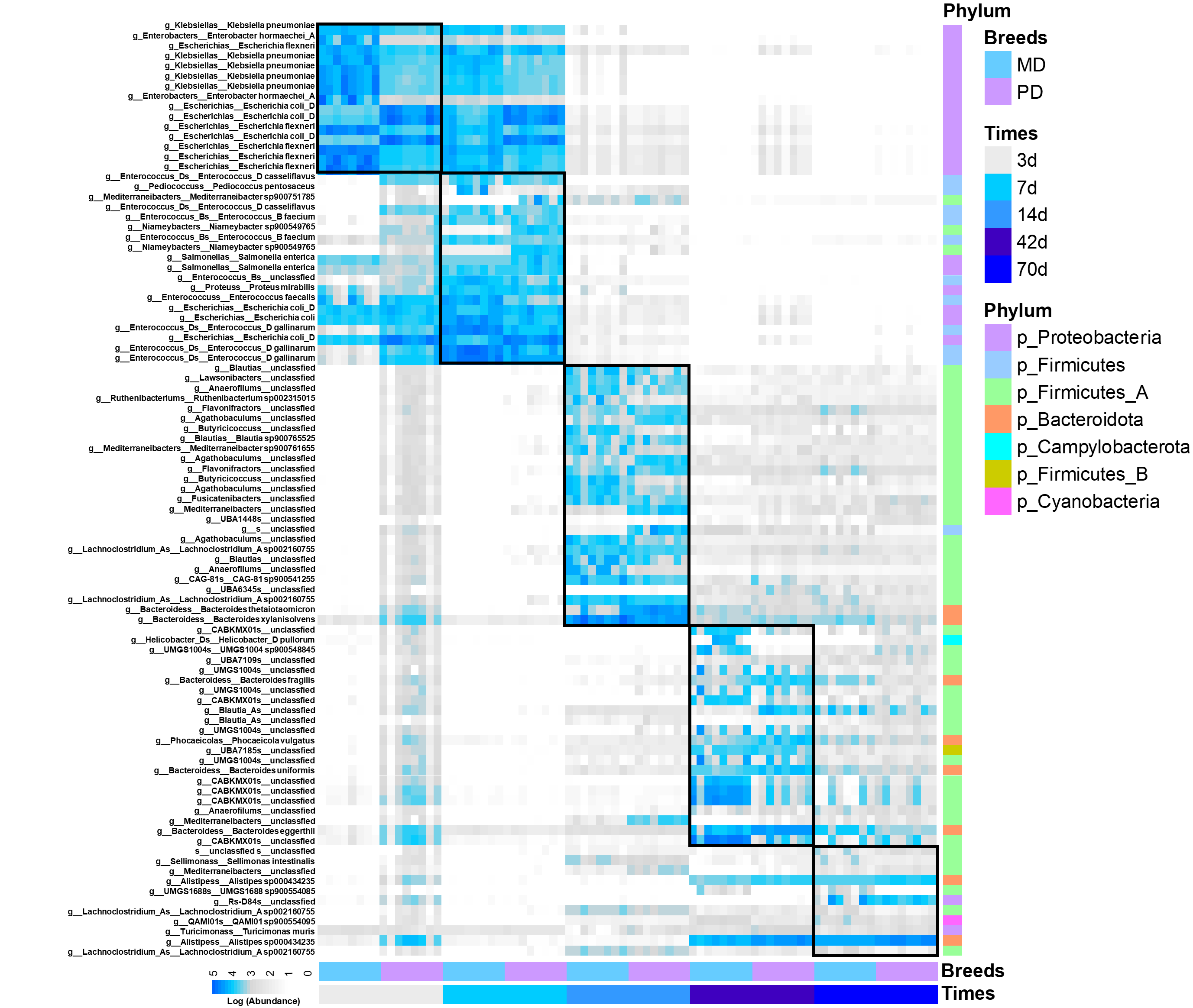


**Figure S7** Heatmap showing stage-associated metagenome-assembled genomes (MAGs) identified by Linear discriminant analysis (LDA) effect size (LEfSe) (LDA>3.5; *p*<0.01) for the Peking duck (PD) and Muscovy duck (MD). The average relative abundances of these MAGs are represented on a log scale.


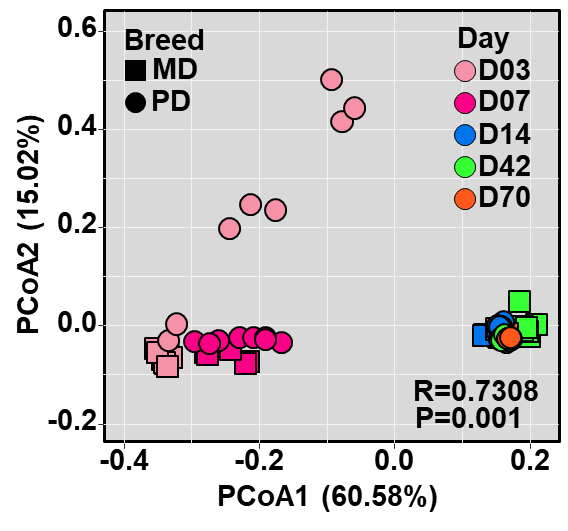


**Figure S8** PCoA analysis based on Bray-Curtis distance of function profiles across growth stages (day 3, 7, 14, 42, and 70).


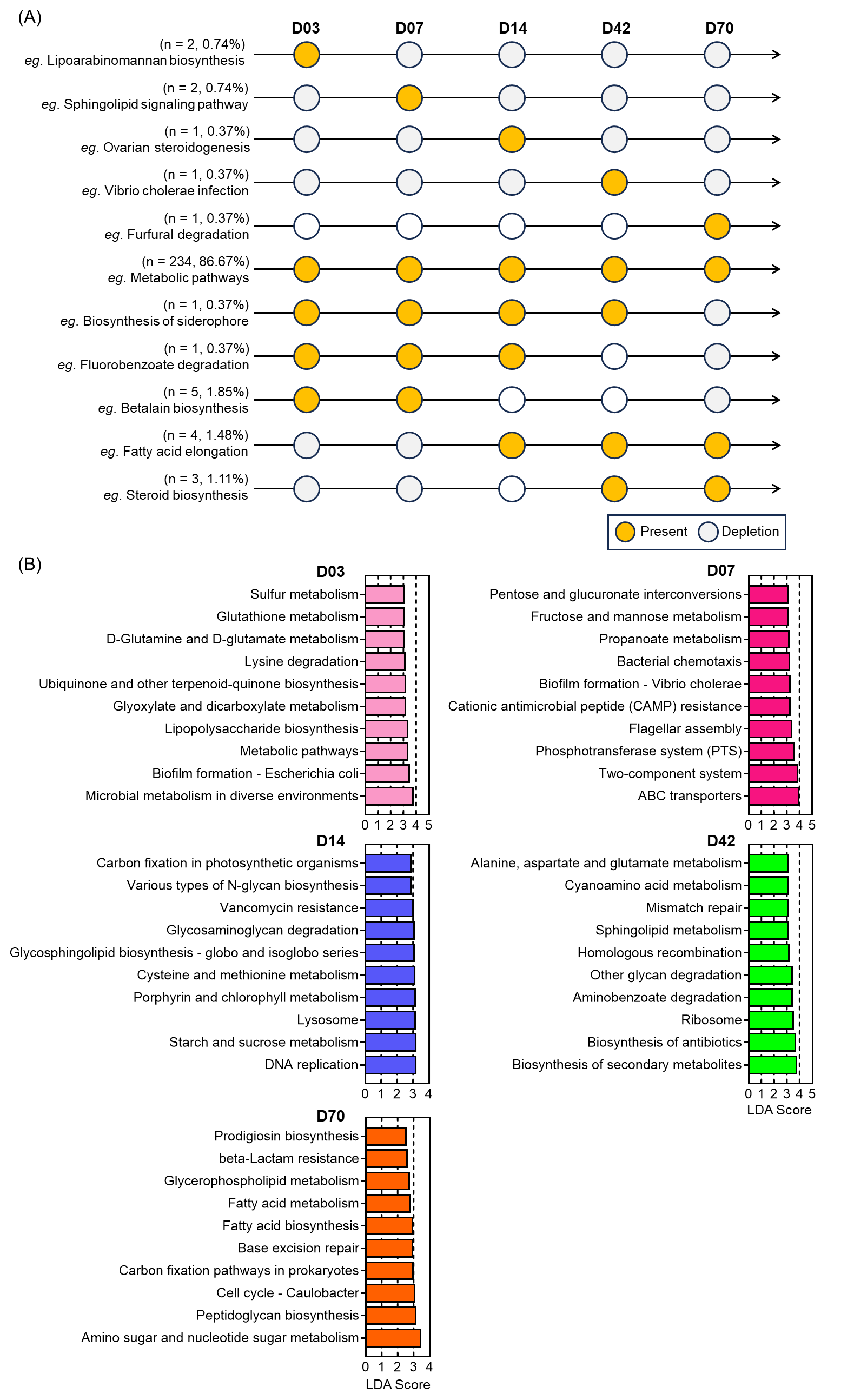


**Figure S9** The microbiome function alteration from ducks at different ages (day 3, 7, 14, 42, and 70). (A) Longitudinal occurrence patterns of the duck gut microbiomes. Total 270 KEGG function based on averaged relative abundance on each day were used to summarize the occurrence patterns. Yellow circle indicates the presence of a bacterial function while a white circle shows the absence. (B) The top 10 KEGG function enriched in the each grow stage.


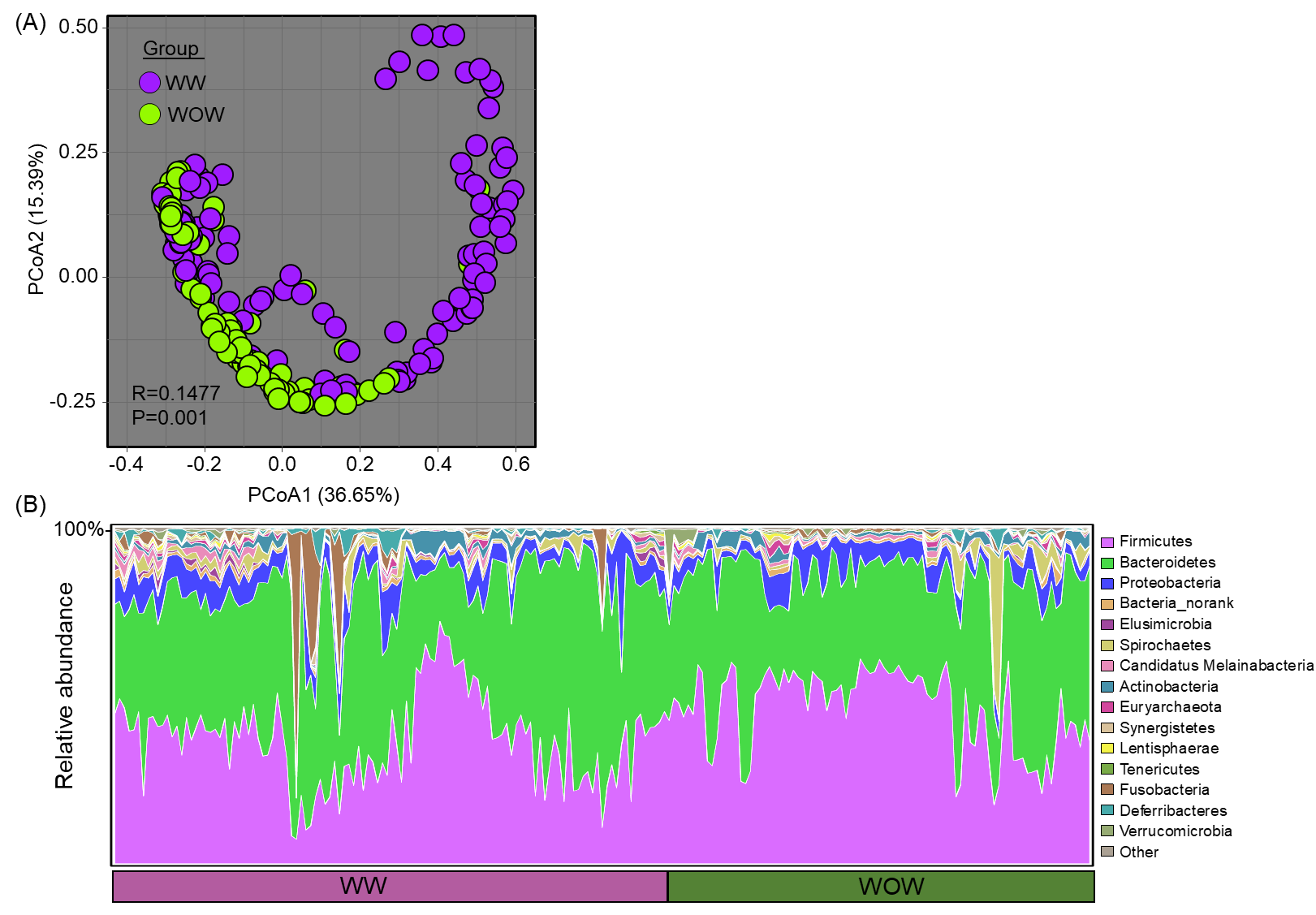


**Figure S10** Duck cecal microbiome changes under different rearing systems. (A) PCoA analysis based on Bray-Curtis distance of taxonomy. (B) Relative abundances of major phyla under the different rearing conditions. WW, with water; WOW, without water.


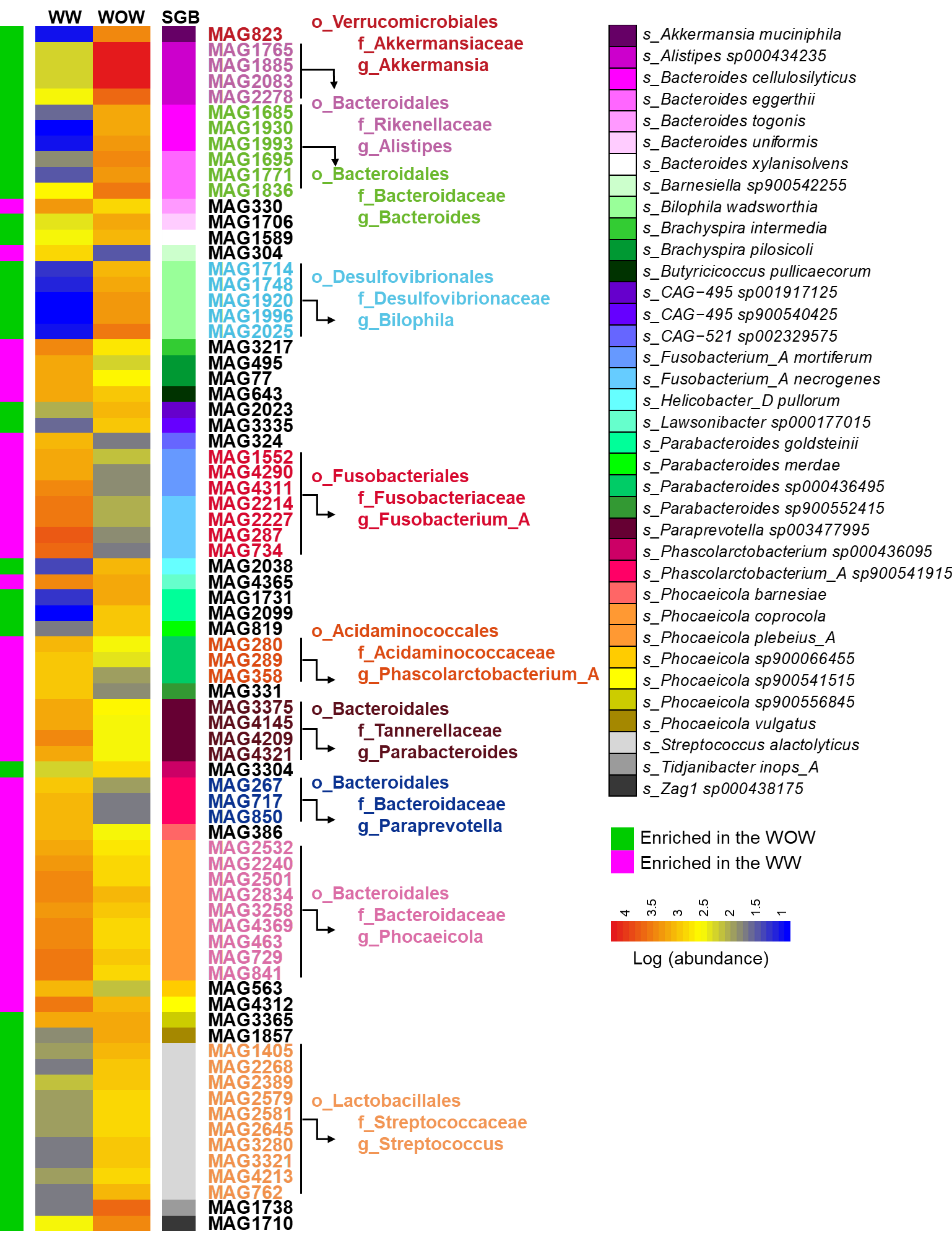


**Figure S11** The species-level genome bins (SGBs) containing metagenome-assembled genomes (MAGs) showing different directions of enrichment in WW and WOW group.


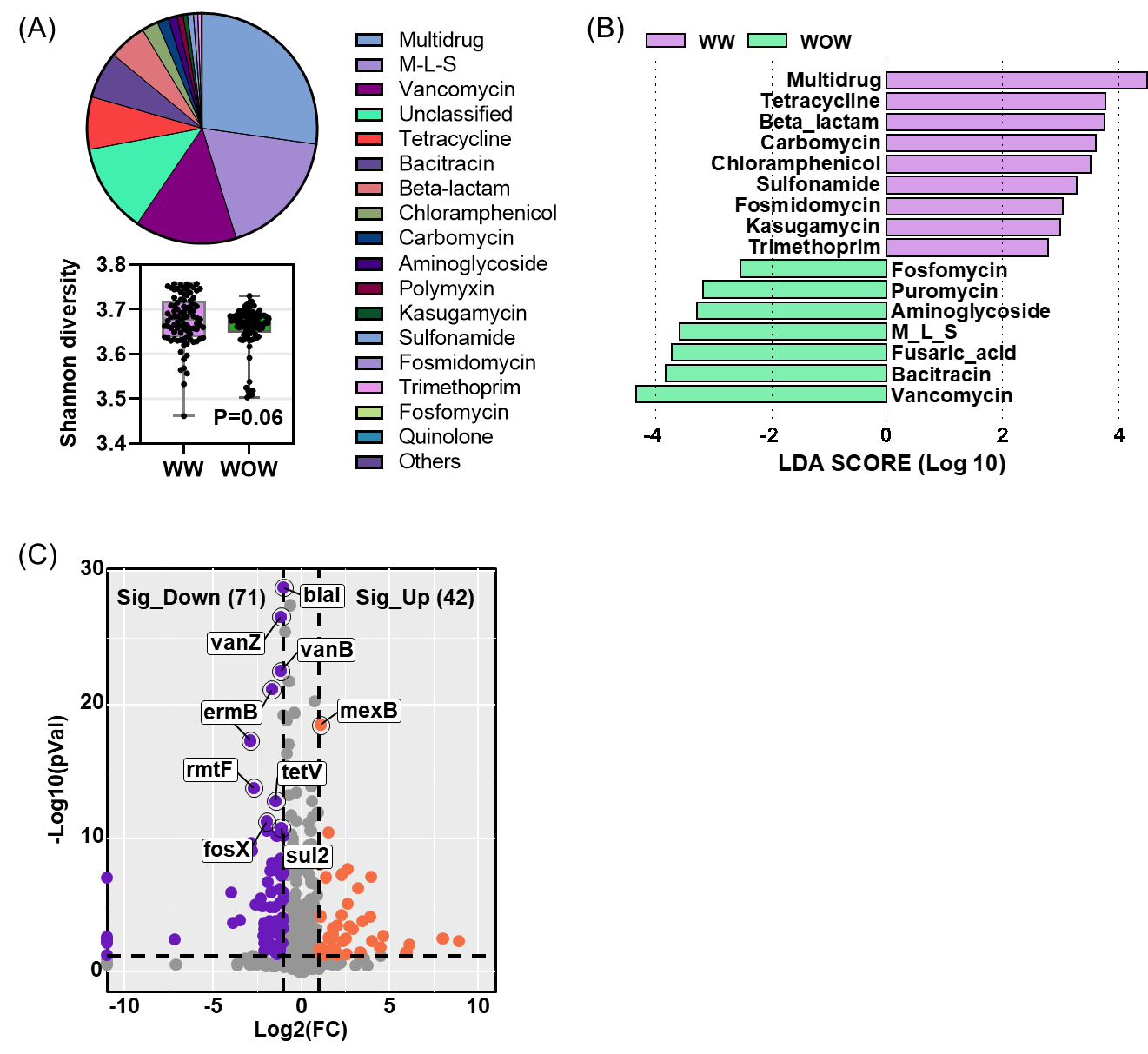


**Figure S12** The antibiotic resistance genes (ARG) profiles under the different rearing conditions. (A) The classification of antibiotic resistance drugs with Shannon diversity in the two groups. (B) Lefse analysis of antibiotic resistance drugs in the two groups. (C) Volcano plot analysis of the difference in ARG subtypes between groups.
